# The role of splenectomy in lipid metabolism and atherosclerosis (AS)

**DOI:** 10.1186/s12944-018-0841-2

**Published:** 2018-08-16

**Authors:** Xiao-Ming Ai, Li-Chen Ho, Lu-Lu Han, Jin-Jing Lu, Xiong Yue, Nian-Yin Yang

**Affiliations:** Department of General Surgery, BenQ Medical Center, The Affiliated BenQ Hospital of Nanjing Medical University, Nanjing, 210019 Jiangsu China

**Keywords:** Splenenctomy, Lipid metabolism, Atherosclerosis

## Abstract

The extensive performance of splenectomy worldwide for patients suffered from splenic trauma has given rise to high risks of postoperative complications, which has been attracting increasing attention in recent years. Nowadays the spleen is regarded as a versatile organ of the human body, invested with various excellent properties. The spleen has been recognized to take a great part in lipid metabolism. While removal of the spleen intends to alter lipid values, especially with an elevated LDL, splenic autotransplantation is able to normalize these lipid alterations. What is more, conservative surgical procedures like subtotal or partial splenectomy, could as well, afford a correction of dyslipidemia. At the same time, clinically, splenectomy demonstrates a high rate of atherosclerosis (AS), whereas non-surgical treatment after splenic trauma shows unchanged propagation of AS. Based on the intimate relationship between serum lipids and AS, the lipid changes modulated by splenectomy are believed to be responsible for the development of AS. Therefore, a “splenic factor” is most likely present in the regulation of lipidation and AS. Several theories have been postulated to elucidate the possible mechanism involved, among which most are primarily based on its forceful natural immune function, that is to say, the mononuclear phagocytic system.However, the accurate mechanisms behind this mysterious phenomenon still remain unclear so far. Of importance, lipid fractions should be monitored consecutively in case of inevitable splenectomy.

## Background

Insufficient knowledge about the spleen had popularized the performance of splenectomy during the past decades, particularly for patients suffered from splenic injuries, until complications ensued gradually one after another in the subsequent years, among which some would be even severe and fatal. The most worrisome is the overwhelming postsplenectomy infection (OPSI), which is characterized as fulminant sepsis caused by encapsulated bacteria [1]. OPSI could occur months, years, even decades later, in particular in children and younger adults, accompanied by a morbidity of approximately 4% and mortality of 2% [2]. Reactive thrombocytosis is another serious concern after splenectomy, showing a reported prevalence of up to 75% [3]. Not only does it directly contribute to venous thromboembolism [3], and more seriously, disseminated intravascular coagulation (DIC), but can it also pose endothelial damage and the subsequent etiology of pulmonary hypertension in the long run [4].

Awakened by the growing awareness of the potential complications following splenectomy, more and more surgeons have paid special attention to the importance of spleen, and have already recognized it as a useful organ of the human body, invested with various excellent properties. To date, it has been well known that the spleen is endowed with powerful filtration, immunological, along with hematopoietic and storage functions [5]. Removal of the spleen would favor a series of dysfunctions [6]. Furthermore, importantly, the spleen takes a great part in metabolic control, which has been attracting increasing attention in recent years, especially in the aspect of lipid metabolism. In general, the spleen takes on a substantial proportion of the organic metabolism and removal of the spleen would obliterate most of the invaluable gifted functions, predisposing to different diseases [7–15]. Apart from the afore-mentioned OPSI and thrombocytosis, asplenism is meanwhile associated with a high risk of vascular events especially as atherosclerosis (AS) and coronary artery disease, diabetes mellitus [16], as well as acute pancreatitis [17] and cancers [18].

### Initial recognition of a splenic influence on lipid metabolism

It has been well established that the spleen is devoted to a wide spectrum of metabolic control, including the metabolism of all metals, albuminoids alongside with indirect bilirubins from senescent erythrocytes [19]. As a matter of fact, the spleen does also participate in lipid metabolism, but our knowledge about it is very limited.

In 1914, King reported an increase in plasmatic cholesterol levels after splenectomy in dogs [20]. Supposedly, there seemed to be a possible splenic influence on cholesterol manipulation. However, it was only many decades later that did this problem begin to attach emphasis. Asai et al. detected increased densities of cholesterols, triglycerides (TG) and phospholipids (PL), in conjunction with decreased HDL after removal of the spleen in rabbits [11]. Thereafter, a great many studies have documented elevated lipid parameters in splenectomized rodent models [9, 12, 21–25], although some of them might have achieved diverse results.

At the same time, while splenectomy tended to alter lipid values in animals, splenic autotransplantation was able to normalize these lipid alterations [21, 23, 25, 26]. Moreover, conservative surgical procedures, like subtotal or partial splenectomy, could as well, afford a correction of dyslipidemia [25–28].

Clinically, the spleen does interfere with the evolution of some diseases associated with lipid disorders. It is the lysosomes of cells of the mononuclear phagocytic system in the spleen that are most frequently present with metabolic changes in times of lipid deposits [27]. Take Gaucher’s disease for example, it is characterized by lipid-loaded cells known as Gaucher cells due to the anomalous accumulation of PL, and is manifested with splenomegaly [27]. Comparable splenic manifestations can also be uncovered in other etiologies like Niemann-Pick’s disease, gangliosidoses as well as Fabry disease [7].

Splenomegaly is inclined to maneuver the cholesterolemia over against splenectomy, which is most likely to develop hypocholesterolemia as a consequence of overfunction of the mononuclear phagocytic system [13]. Myeloproliferative disorders like polycythemia vera and myelofibrosis, are usually accompanied by hypersplenism and splenomegaly, displaying a reduction of serum cholesterol profiles [27]. By comparism, splenectomy is capable of reversing these cholesterol alterations [8, 9, 13, 14]. Moreover, in other diseases such as Gaucher’s disease [15] and type B thalassemia major [10], similar results could be obtained likewise.

**Fig. 1:**
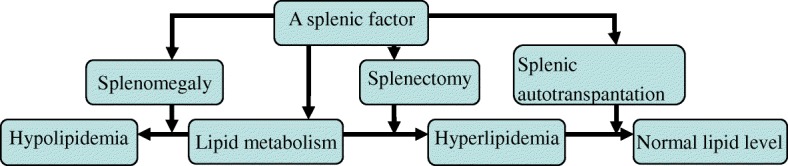
a flow diagram illustrating a splenic factor in lipid metabolism

In light of these available findings, we have reasons to believe that the spleen participates in lipid metabolism to an extreme extent, although lack of adequate convincing evidences. Therefore, a “splenic factor” proposed by Asai et al. [12] is most likely present in the regulation of lipidation, just as illustrated in Fig. 1.

### Initiate recognition of the impact of splenectomy on lipid metabolism and AS

AS is the leading cause of cardiovascular disease (CVD), which most of the time features acute heart attack and stroke [29], and accounts for the soaring morbidity and mortality worldwide [30]. The pathogenesis of AS is very complex, involving a variety of risk factors and mediators, among which the circulating cholesterol concentrations remain as the most important ones, especially LDL and HDL [29]. In fact, elevated serum TG and LDL, together with reduced HDL, are well identified as the atherogenic lipid triad [27]. Generally speaking, AS is a metabolic disease in essence, which is, as a result of lipid disorders, characterized by lipid deposition in the intimal layer of arteries as well as atheromatous plaque formation [10].

In 1977, the long-term observation of veterans subjected to tramatic splenectomy during World War II disclosed an intensive susceptibility to coronary atherosclerotic heart disease [31]. Based on the close relationship between serum lipids and AS, the lipidic metabolic changes modulated by splenectomy were believed to be responsible for the rising mortality due to acute myocardial stroke [9, 32], thus leading to the essential investigations of the possible modulating role of spleen in lipid disorders and AS.

As expected, it seemed extremely evident in animals [10, 12, 22, 25, 33] that removal of the spleen worsened lipid metabolism and accelerated AS. Clinically, patients submitted to traumatic splenectomy demonstrated a high rate of hyperlipidemia together with AS or acute myocardial infarction [9, 25, 34, 35]. On the contrary, while in individuals suffered from hereditary spherocytosis (HS), splenenctomy exhibited high atherosclerotic lesions [35], a preserved spleen could allow low adverse arterial events [36]. In the meantime, patients dealed with non-surgical treatment after splenic trauma showed unchanged propagation of AS [34].

Therefore, it is justified to speculate that, the absence of spleen apparently determines the occurrence of AS on account of deterioration of serum lipids, indicating a protective splenic effects [33].

### Results of determination of serum lipid concentrations upon splenectomy

A number of studies have been carried out to evaluate influence of the spleen in lipid manipulation, which however, have created different and controversial results, with a vast preponderance of elevated lipid parameters by splenectomy.

Experimentally, the majority of studies have provided evidences of increased concentrations of TG, TC, LDL, and decreased HDL, in combination with promotion of atherosclerotic changes in the absence of spleen [9, 12, 13, 23, 25, 27], although with some variations, which alleged no alterations in TG or VLDL fractions [13, 25, 27]. Nonetheless, there are exceptions. Some researchers peculiarly pointed out an elevation of all lipid parameters upon removal of the spleen [22, 26, 27]. In contrast, some others proved no abnormaly in LDL regardless of the increment in other lipid fractions [12, 23]. What is more, some else even reported a declining tendency of LDL in opposition to the rise of other fractions [37].

Clinically, splenectomy is equally coupled with a capability of modifying lipid values, especially with an elevated LDL [13, 14, 28].

On the other hand, apart from the above-mentioned lipid disturbances, there are even discrepant outcomes. Some study revealed reduced LDL, and increased HDL [37], whereas others didn’t detect any significant changes in lipid parameters [10, 38–40], among which some exhibited enhanced atherosclerotic lesions [10, 38, 39], and the remaining was even in favor of attenuated coronary artery atherosclerosis [40]. Furthermore, there are still few studies reflecting no abnormalies in plasmatic lipid indices and atherogenesis at all [24, 41].

Therefore, the role of spleen in lipid metabolism and AS still remains debated, irrespective of the strong supportive evidences.

### Effect of diet in lipid control

As in human being, a diet is the first-line management for plasmatic lipid levels and cardiovascular disease [42], it could also exert a similar action in lipid control in splenectomized animals despite of lack of appropriate clinical trials [41], and in this respect, fatty components in a diet could be more effective than cholesterol [30]. Researches have verified that the types of chow were able to augment the alterations in lipid profiles by splenenctomy [9, 26].

Once again, some other voices arise representing different standpoints. An author argued that worsening of lipid fractions was entirely ascribed to the quality of chow other than splenectomy in rats [41]. In contrast, another one insisted that the very lipidic abnormaly was intrinsically relevant to the spleen, not the diet at all [32]. Hence, dissents will continue within this context.

### The possible mechanisms

The plasma lipids are fats or complex fat-like molecules, mainly referring to triglycerides, cholesterols, as well as phospholipids (PL) and free fatty acids (FFA). In brief, the process of lipid metabolism encompasses digestion and absorption in the small intestine, transportation within lipoproteins through the blood, and then biosynthesis in the liver. It is extremely intricate and subtle, requiring the help of a great deal digestive enzymes, and the liver is the pivotal organ during this course. Trouble in any steps of this process is likely to bring about lipid metabolism disorders, which are associated with an increase in the concentrations of plasma lipids.

Among the numerous studies focused on dyslipidemia upon splenectomy, very few conferred deep insight into the mechanism implicated in lipid regulation by the spleen, thus further acting on the development of AS. There is still a long way for us to completely unveil its mysterious piths. Several theories have been postulated to elucidate the possible mechanism, among which most are primarily based on its forceful natural immune function, in other words, the mononuclear phagocytic system. But none of them have been well accepted. On the other hand, a number of potential predisposing factors different from lipid dysfunctions, like disturbance in coagulation, abnormal macrophage involvement in the plaque [40], in combination with some other factors, could be also validated in the regulation process. Thence, no consensus has been attained so far.

#### Lipid reservoir

The most common and popular explanation was initially proposed by Schmidt et al., comparing the spleen to a storage reservoir for lipids [8]. In case of hypersplenism, the splenic macrophages accumulate a large proportion of fat through hyperactive phagocytosis, resulting in hypolipidemia, while splenectomy can engender an inverse effect, provoking reversal in serum lipid densities [37]. Accordingly, it is well known as a splenic factor involved in lipid control (as shown in Fig. 1), suggested by Asai et al. [12]. This theory seemingly covers both the macrophage activity and the available splenic volume. In states of splenomegaly, overloaded splenic tissue exaggerates the impact of the splenic factor by phagocytosis, with dramatically rising storage volume for lipid, thus lowering serum lipid values [43], whereas in an asplenic or hyposplenic condition, this very mentioned factor is missed, the splenic parenchyma as well as the macrophage activity undergoes destruction, and the metabolic reactions meet with repercussion, thus improving lipid parameters [27]. However, this theory is unable to fully interpret the currently existent evidences of lipidic metabolic changes in splenic cells, thus necessitating an in-depth investigation into its innate cellular function.

#### The mononuclear phagocyte system against lipid fractions

From a metabolic point of view, the spleen has been established as an important site of LDL catabolism by the efficient macrophage activity [31]. It has been hypothesized that in the event of splenomegaly, hypocholesterolemia would take place through amplified LDL catabolism, as a result of overfunction of macrophage activity [44]. According to this theory, in the presence of the spleen, this instinctive auto-immune effect imposes on the structures of lipoproteins, extraordinarily HDL and LDL, giving rise to their plasmatic purification and conversion into foamy cells [10, 15, 33], which is accompanied by hypocholesterolemia. Conversely, in the absence of the spleen, lack of macrophage impairs the clearance mechanism, leading to the corresponding reduced catabolism together with the attendant hypercholesterolemia [22].

Noticeably, it needs to figure out that the liver is the principle organ in view of lipoprotein synthesis and excretion. The changes in lipid parameters by splenectomy are more likely to originate from exaggerated hepatic production of triglyceride-rich lipoproteins rather than attenuated catabolism, which would be more eligible to take charge of a decrement in HDL levels [9]. In parallel with this assumption, upon removal of the spleen, a reinforced LDL receptor (LDLR) activity is induced to counteract the attenuated LDL catabolism [9]. By binding to LDLR, LDL-LDLR complex is then endocytosed into the liver Kuppfer cells for degradation, and afterwards, LDLR would return to the cell surface for recirculation [45]. However, as another distinguished major lipid pathway [45], the degradation products may later on, be transported back to the liver for reutilization which are subsequently refabricated for relipidation, leading to a resultant augmented cholesterol load in hepatocytes [9]. As a consequence, synthesis of triglyceride-rich lipoproteins is augmented, especially as LDL, which in reverse, with the help of cholesteryl ester transfer protein (CETP) [46, 47], are able to propel the final selective removal of nascent HDL by the liver, leading to lessened HDL densities [48]. Eventually, an exaggerated LDL in company with lessened HDL exacerbates atherogenensis.

Needless to say, the production along with purification of lipoproteins modulated by the spleen, is an extremely sophisticated course, and during this course, a variety of mediators may be drawn into. For instance, ATP-binding cassette (ABC) transporter ABCA1 favors macrophage cholesterol efflux to lipid-poor apolipoproteins like apoA-I [49], thus facilitating the production of HDL [50]. Deficiency of ABCA1 would accelerate atherosclerotic lesion development [51]. But whether ABCA1 plays a positive or negative influence on lipid control still remains unclear. Cholesteryl ester transfer protein (CETP) is responsible for the exchange of cholesteryl esters (CE) and TG between apoB-containing lipoproteins and HDL [46, 47], in parallel with an increment in LDL and decrement in HDL.

#### Anti-oxLDL antibodies

Authentically, AS has been declared to arise from vascular inflammation coupled with dyslipidemia, and LDL has been well considered as a risk factor for AS. Deposits of LDL in the arterial wall would aggravate the formation of atheromatous plaque. According to the oxidative theory [52], deposits of LDL as well as the subsequent oxidative modifications in the LDL molecules, pose damage to the vascular endothelium [52], initiating the atherosclerotic scenario. And a comprehensive interplay of the modified lipoproteins, such as oxidized LDL (oxLDL), macrophages, lymphocytes, in combination with normal cellular constituents of the arterial wall [52], perpetuates the progression of this disease. The atherosclerotic lesion attributes specific immune responses to oxLDL and other antigens [38].

The spleen has been established to be the staple organ responding to blood antigens, specifically to the oxLDL [10, 33, 38], stimulating the production of anti-oxLDL antibodies intermediated by B lymphocytes [28, 38, 53]. Based on this highly regulated phenomenon, some scholars would like to take advange of these antibodies to clarify a potential antibody-type protection against AS [33, 38]. The immune activity of circulating antibodies to oxLDL is able to evoke internalization of oxLDL into macrophages and its ultimate purification. By comparison, splenectomy is disposed to elimination of this valuable immune capability, giving rise to reduction of the oxLDL clearance and the subsequent aggravation of AS. Remarkably, experimental reports have indicated the association between an increment in anti-oxLDL antibody titers and a decrement in atherosclerotic lesions [33, 38].

By this token, the spleen is not only dedicated to the production of anti-oxLDL antibodies, but also to the clearance of antigen-antibody complexes [43]. However, unexpectedly, there are also few documentations affirming exacerbation of atherosclerotic lesions with no differences in serum titers of anti-oxLDL antibodies after splenectomy [10, 39]. Hence, the role of these antibodies in AS still has not been confirmed thus far [53], and what is more, it is not sensible to simply accredit the spleen’s participation in AS to the production of these antibodies [10].

#### Involvement of lipidic activities of the liver

The spleen may also interfere with the lipidic metabolic change of the liver on account of decreased lipid peroxidation following splenectomy [22]. As far as lipidic genesis, lipoproteins are mainly assembled and excreted in the liver and small intestines, exporting lipids (cholesterol, TG and PL) to the circulation system and in turn, importing excessive lipids to the liver for clearance or recycling [45]. The liver possesses enormous amounts of Kupffer cells, which primarily account for elimination of bacteria in conjunction with engulfment and clearance of lipoproteins via peroxidatic reaction [22]. Of note, splenectomy is liable to inhibit the responsiveness of Kupffer cells [54], thus bringing about dyslipidemia.

In addition, LDL receptors (LDLR) also partake in the modulation of dyslipidemia upon splenectomy. Upon removal of the spleen, enhanced LDLR activities are elicited in the liver [9], as mentioned above. It is believed that, LDLs, which transport most cholesterol, bind to LDLRs first, and then are uptaken and cleared in the liver or small intestine [45]. Just as anticipated, enhanced LDLR activities exacerbate LDLs clearance. Besides, chylomicron together with VLDL remnants are also swept away by specific liver receptors [55]. Thereby, there is an argument that disturbance of lipoprotein receptors in the liver may be as well in charge of dyslipidemia after splenectomy [9].

#### Lipoprotein lipase (LPL)

LPL, as an enzyme responsible for the transportation and storage of lipids as well as hydrolyzation of chylomicrons and TGs, is most active in the hepatocytes. It can be stimulated by ApoC-II and suppressed by ApoC-III [56]. Amazingly, LPL activity has been also found in the spleen. Under the condition of splenomegaly, high LPL activity has been testified to accelerate cholesterol deposition [57], thus offering an additional proof which supports the theory taking the spleen as a storage reservoir.

#### Shifts in MicroRNAs (miRNAs) expression

New-emerging disciplines on the noncoding sequences of miRNAs have corroborated their functions as the posttranscriptional regulation of gene expressions [58]. miRNAs have also been identified as pivotal regulators for lipid metabolism in recent years, playing an influence in AS [59]. Purportedly, a body of miRNAs have been proved to regulate genes correlated with HDL metabolism, especially miR- 33a/b [60], which may contribute to ABCA1 expression at the same time, while many others, such as miR-148a, miR-128-1, or miR-30c, are involved in controlling circulating LDL concentrations as well as hepatic LDLR expressions [61]. Shifts in miRNAs expression after splenectomy may possibly take part in the development of AS [62]. But the accurate mechanism remains still unclear.

#### The platelet (PLT) pathway

It is a very common phenomenon that PLT counts experience uprising upon splenectomy, which could otherwise be reverted under the conditions of splenic autotransplantation [22, 27]. It has been advocated that splenectomy may accelerate the progression of AS through the PLT pathway [40], independent of lipid dysfunction [9, 25]. Thrombocytosis in combination with hypercoagulability is prone to PLT accumulation and aggregation [9], and the subsequent thrombus formation within the arterial wall, acting as a causative factor for AS [9].

#### Immune-mediated mechanism

Needless to say, the spleen is a multipurpose organ, massively synthesizing and accommodating large quantities of granulocytes, megakaryocytes, alongside with lymphocytes and macrophages, which have been verified to make great contributions to the process of AS. It is the major organ obligated to lymphocytes in the human body, and assumes the duties of maintenance of the immune system, by regulating both local and systemic immunity [63].

Reportedly, a predominant number of memory B cells are pooled herein, which following activation, are apt to protect against atherosclerotic lesion formation [64], most probably through mitigation of the characteristic proinflammatory cytokine response of the T-helper 1 (Th-1) [65]. Large amounts of regulatory T cells (Tregs) are also harbored in the spleen, which can similarly repress AS [66], accompanied by biogenesis of a battery of other biomolecules [38]. Meanwhile, Th2 cells classically cultivated in the spleen, are mediated by B cells and antibodies, and substantially inhibit effects of Th1 cells [10], thus exerting protective impact on AS.

On the contrary, Th1 lymphocytes, which are responsible for IFN-γ production and macrophage activation [67], in conjunction with the activated macrophages, have already been attested to aggravate the atherogenesis throughout the whole process [68].

Taken altogether, it has even been declared that, following removal of the spleen, an integrated combination of macrophages, T cells together with an imbalance between the Th1/Th2 patterns [64], B cells, as well as their recruitments at the lesion site, is hypothesized to exclusively implement the mechanism, precluding any lipidic activities. Moreover, some authors have already observed atherogenic deterioration lack of serum lipid abnormalities in splenectomized rodent models, thus suggesting an immune-mediated mechanism, other than modulation of lipidation [10].

#### Adverse viewpoints

In contrast, certain explanations appear likewise plausible with respect to improvement of AS by splenectomy. Reportedly, the spleen is regarded as a key manufacturer for monocytes and the most critical section of the mononuclear phagocyte system. It has stated that in the setting of acute myocardial infarction, the spleen is capable of rapidly mobilizing large quantities of mononuclear phagocytes directly onto the endothelium in the vessel wall in response to acute inflammation [69]. Moreover, they can also trigger the massive macrophage recruitment in fatty streak lesions [70], thus introducing another contributing factor to AS. However, removal of the spleen would cut down circulating monocyte concentrations, giving rise to fewer monocytes arrest in the aortic plaque [40]. Ultimately, a shrunken inflammation could improve the atherosclerotic condition.

In addition, a characteristic infiltration of macrophages in the vascular tissue could be found in response to lipid deposited in the atherosclerotic lesions [40]. Theoretically, asplenism is linked with a decrement of macrophage densities. Nevertheless, exceptions arise once more. Some research has displayed mitigation of AS lacking decline in macrophage densities after removal of the spleen [40], but with modified distribution of macrophages across the plaque, media, and adventitia [40], which was also consistent with some other studies [71].

## Conclusions

In view of the conflicting results alongside with the obscure mechanism, contradictions will persist in the future as regards the role of splenectomy in lipid control and AS, and more relevant experimental and clinical studies will be needed. With the growing recognition of the spleen being an important organ with versatile functions apart from its impact on metabolic control, multiple modalities of spleen-conserving procedures such as vascular occlusions, splenorrhaphies, as well as partial splenectomies, have been utilized in recent years, so as to preserve the splenic tissues. Splenic tissue autotransplantation is an effective approach to partially reconstitute its structure and function in case of unavoidable splenenctomy, which is substantially capable of restoring the lipid and platelet (PLT) parameters [22, 27]. Furthermore, it is worth noting that the non-surgical treatment for splenic trauma tends to gain better outcomes than surgery [72].

On the other hand, it should be admitted that this very role of splenectomy in dylipidemia and AS may be highly regulated, albeit extremely intricate. It involves probably the synergistic interactions between nutritional condition, immunologic response, as well as metabolic control and coagulation, and is in the meantime influenced by numerous other factors, such as demographics, poor diet, sedentary lifestyle, along with hormonal and genetic predisposition. Thereby, it is indeed problematic and challenging for clinicians to deal with, and lipid fractions should be monitored consecutively in patients subjected to splenectomy.

## Abbreviations

ABCA1: ATP-binding cassette (ABC) transporter
AS: atherosclerosis
CE: cholesteryl esters
CETP: cholesteryl ester transfer protein
CVD: cardiovascular disease
DIC: disseminated intravascular coagulation
FFA: free fatty acids
LDLR: LDL receptor
LPL: lipoprotein lipase
miRNAs: MicroRNAs
OPSI: overwhelming postsplenectomy infection
oxLDL: oxidized LDL
PL: phospholipids
PLT: platelet
TG: triglycerides
Th-1: T-helper 1
Tregs: regulatory T cells
